# Foraging choices of vampire bats in diverse landscapes: potential implications for land‐use change and disease transmission

**DOI:** 10.1111/1365-2664.12690

**Published:** 2016-05-26

**Authors:** Daniel G. Streicker, Jacob E. Allgeier

**Affiliations:** ^1^ Institute of Biodiversity, Animal Health and Comparative Medicine University of Glasgow Glasgow G12 8QQ United Kingdom; ^2^ Medical Research Council‐University of Glasgow Centre for Virus Research Glasgow G61 1QH United Kingdom; ^3^ Odum School of Ecology University of Georgia 140 East Green St. Athens GA 30602 USA; ^4^ School of Aquatic and Fisheries Science University of Washington Seattle WA USA

**Keywords:** anthropogenic change, Chiroptera, disease transmission, food web, foraging behaviour, neotropics, rabies virus, vampire bat *Desmodus rotundus*, zoonotic, zooprophylaxis

## Abstract

1. In Latin America, the common vampire bat *Desmodus rotundus* is the primary reservoir of rabies, a zoonotic virus that kills thousands of livestock annually and causes sporadic and lethal human rabies outbreaks. The proliferation of livestock provides an abundant food resource for this obligate blood‐feeding species that could alter its foraging behaviour and rabies transmission, but poor understanding of the dietary plasticity of vampire bats limits understanding of how livestock influences rabies risk.
2. We analysed individual‐ and population‐level foraging behaviour by applying δ^13^C and δ^15^N stable isotope analysis to hair samples from 183 vampire bats captured from nine colonies in Peru. We also assessed the isotopic distributions of realized prey by analysing blood meals extracted from engorged bats and samples collected from potential prey species. In two adjacent but contrasting areas of the Amazon with scarce and abundant livestock, we used questionnaires to evaluate the incidence of feeding on humans.
3. Population‐level isotopic signatures suggested substantial among‐site variation in feeding behaviour, including reliance on livestock in some colonies and feeding on combinations of domestic and wild prey in others. Isotopic heterogeneity within bat colonies was among the largest recorded in vertebrate populations, indicating that individuals consistently fed on distinct prey resources and across distinct trophic levels. In some sites, isotopic values of realized prey spanned broad ranges, suggesting that bats with intermediate isotopic values could plausibly be dietary specialists rather than generalists.
4. Bayesian estimates of isotopic niche width varied up to ninefold among colonies and were maximized where wildlife and livestock were present at low levels, but declined with greater availability of livestock. In the Amazon, the absence of livestock was associated with feeding on humans and wildlife.
5. *Policy implications*. We provide the first insights into the foraging behaviour of vampire bats in habitats with common depredation on humans and show how vampire bat foraging may respond to land‐use change. Our results demonstrate risks of rabies transmission from bats to other wildlife and are consistent with the hypothesis that introducing livestock might reduce the burden of human rabies in high‐risk communities.

## Abstract 2

We provide the first insights into the foraging behaviour of vampire bats in habitats with common depredation on humans and show how vampire bat foraging may respond to land‐use change. Our results demonstrate risks of rabies transmission from bats to other wildlife and are consistent with the hypothesis that introducing livestock might reduce the burden of human rabies in high‐risk communities.

## Introduction

Land‐use changes such as urbanization and agricultural intensification alter the dietary resources available to wildlife (Oro *et al*. 2013). The ability to exploit these resources can influence whether species thrive or decline following environmental change (Sih, Ferrari & Harris 2011). The resulting shifts in population dynamics and community composition can affect a variety of ecosystem processes including food web structure, dispersal and energy flow (Jefferies, Rockwell & Abraham 2004; Layman *et al*. 2007b). Resource shifts can also have important consequences for the transmission of infectious diseases by altering demographic processes, animal interactions and host immunity (Becker, Streicker & Altizer 2015).

The common vampire bat *Desmodus rotundus*, an obligate blood‐feeding species, has experienced changing availability of wild and domestic prey throughout its range from Mexico to northern Argentina. Intensification of livestock rearing has created a novel, abundant and reliable source of blood that has caused population growth and geographic range expansions (Delpietro, Marchevsky & Simonetti 1992; Lee, Papes & Van Den Bussche 2012). These changes have direct implications for human and animal health because *D. rotundus* is the most important reservoir of *Rabies virus* in Latin America – a lethal zoonotic disease that kills thousands of livestock annually and causes recurrent human rabies outbreaks in the Amazon rain forest (Schneider *et al*. 2009). Direct costs from livestock mortality (i.e. before costs of human and domestic animal vaccination, laboratory diagnostics and bat control) exceed US$30 million per year (WHO 2013). Rabies is transmitted from vampire bats to other species when bats bite to feed on blood; thus, understanding how changing environments affect bats’ foraging decisions provides a link between food web and disease ecology.

Past studies have used stable isotope analysis and molecular typing of DNA in vampire bat faeces to demonstrate reliance on livestock when they are locally abundant (Voigt & Kelm 2006; Bobrowiec, Lemes & Gribel 2015). Feeding habits in regions without livestock are poorly characterized (Catenazzi & Donnelly 2008), but critically, it is these areas where bat bites on humans are common, representing a serious public health risk (Schneider *et al*. 2009; Stoner‐Duncan, Streicker & Tedeschi 2014). Here, we address several unanswered questions about vampire bat foraging that have implications for human and animal health. First, do individuals within the same colonies specialize on distinct prey? Vampire bats have been hypothesized to repeatedly forage on the same individuals, suggesting the capacity for specialization (Greenhall 1988). However, most previous studies were conducted where domestic prey are plentiful so tendencies towards specialization in regions with diverse wildlife prey and few livestock are unknown (Voigt & Kelm 2006; Bobrowiec, Lemes & Gribel 2015). If specialization occurs, then only certain individuals may be responsible for human or livestock rabies and control programmes might target these individuals. Secondly, does the introduction of livestock homogenize or add complexity to the diet of vampire bats at the population level? Homogenization could result from a total switch from native prey to livestock, while heterogeneity could increase if only a subset of the colony utilize livestock. Thirdly, will introduction of livestock to forested areas reduce vampire bat depredation on wildlife and humans? As local removal of livestock has been associated with greater depredation on humans (McCarthy 1989), we hypothesize that bat bites on humans could decline when livestock are introduced.

Stable isotopes (δ^13^C and δ^15^N) are a useful tool for studying diet because they integrate prey choices across long time spans when quantified from tissues with slow turnover such as hair (Peterson & Fry 1987), which in vampire bats represents diet averaged over 4–6 months prior to sampling (Voigt & Kelm 2006). If two individuals from the same population have different isotopic signatures in their hair, this implies (i) diversity in the isotopic values of available prey, and (ii) the individuals must have fed on isotopically distinct subsets of the available prey community over prolonged time‐scales (Layman *et al*. 2012). The relative spread of individuals within the same population across isotopic space can be quantified as isotopic niche width and compared among populations (Bearhop *et al*. 2004; Layman *et al*. 2007a). Assessing the isotopic distributions of realized prey (e.g. stomach contents) can help differentiate whether variation in isotopic niche width reflects different prey availability to each population (i.e. absence of prey items with high or low isotopic signatures) or different foraging behaviour with the same prey available (Araújo *et al*. 2009) and provides insights into the consistency of individuals’ feeding preferences through time (Voigt & Kelm 2006).

To explore effects of livestock on vampire bat foraging patterns, we quantified population niche width in vampire bat colonies across three ecoregions of Peru: the Amazon, the Andes and the Pacific coast. Within each ecoregion, we sampled bat colonies with different access to domestic and wild prey to determine how the presence or absence of isotopically distinctive species influenced bat foraging behaviour and isotopic niche width. Within colonies, we compared isotopic values in hair (representing long time spans) and vampire bat blood meals (representing contemporary feeding) to evaluate the consistency of feeding preferences through time. As a case study of how introduction of livestock into the rain forest alters vampire bat foraging and human rabies risk, we conducted oral questionnaires on patterns of bat–human contact in two neighbouring sites in the Amazon undergoing either intensification of gold mining (in the near absence of livestock) or intensification of livestock production associated with the completion of the Interoceanic highway. Importantly, this represents the first window of insight into the feeding behaviour of bats in habitats of high risk for zoonotic disease transmission where depredation on humans is common and provides an opportunity to ask how dietary niche width responds to the introduction of livestock.

## Materials and methods

### Capture and sampling of vampire bats and sympatric species

We captured vampire bats from nine colonies in Peru between April 2008 and March 2013 in the departments of Lima (hereafter coast, *N* = 3 colonies), Apurímac and Cajamarca (hereafter Andes, *N* = 3 colonies), and Madre de Dios and Amazonas (hereafter Amazon, *N* = 3 colonies; Table 1). Sites were daytime roost structures (caves, mines, tunnels and hollow trees) to avoid biasing dietary inferences by sampling bats near potential prey while foraging (Bobrowiec, Lemes & Gribel 2015). Bats were captured using mist nets and/or a harp trap placed outside of each roost between 18:00 h and 06:00 h. Age was classified as juvenile or adult based on fusion of the phalangeal epiphyses (Anthony 1988). For stable isotope analysis, we trimmed ~0·4 mg of hair from the dorsal posterior region of each bat and stored samples on ice packs until they could be frozen (typically 0–2 days). We analysed 14–37 individuals per colony (average = 20·3; Table 1, Appendix S1, Supporting information).

**Table 1:** Sampling localities of vampire bats in Peru

Site	Department	Ecoregion	Latitude	Longitude	Elevation (m)	Roost type	Bats sampled	Livestock^b^
L‐10	Lima	Coast	−11·59	−77·28	16	Tunnel	14	Cow, pig
L‐6	Lima	Coast	−11·06	−77·46	307	Tunnel	14	Cow^a^, donkey^a^
L‐4	Lima	Coast	−13·59	−73·45	15	Mine	23	None
A‐1	Apurimac	Andes	−13·45	−73·83	1971	Cave	14	Goat^a^
A‐9	Apurimac	Andes	−13·59	−73·35	3013	Cave	14	Cow^a^, donkey^a^, horse
C‐4	Cajamarca	Andes	−5·17	−78·95	924	Cave	23	Cow^a^
M‐134	Madre de Dios	Amazon	−13·05	−70·37	304	Tree	37	Cow^a^
M‐130	Madre de Dios	Amazon	−13·01	−70·65	309	Tree	21	Pig^a^, chicken^a^, dog, cat,
AM‐2	Amazonas	Amazon	−5·22	−78·28	329	Cave	23	Cow^a^

a Species observed bitten by vampire bats.

b cow=*Bos taurus*, donkey=*Equus africanus asinus*, goat=*Capra aegagrus hircus*, horse=*Equus ferus caballus*, pig=*Sus scrofa domesticus*.

To characterize the isotopic distribution of prey species and compare integrated feeding patterns from bat hair to a snapshot of feeding on the night of capture, we developed a non‐lethal procedure to collect stomach contents from bats that were captured after feeding. Bats were anaesthetized by intramuscular injection of ketamine (8·3–12·5 mg kg^−1^), and a sterile 5‐French nasogastric feeding tube was inserted to the stomach via the oesophagus and attached to an empty syringe. Extracted blood (ca. 50 μL) was expelled onto Whatman Flinders Technology Associates (FTA) cards and desiccated. We collected 65 blood meal samples from 65 individual bats, from eight of the nine sites (A‐1 = 8, A‐9 = 2, C‐4 = 2, L‐10 = 5, L‐4 = 9, L‐6 = 17, M‐134 = 13, M‐130 = 12; Appendix S2). Bats were stored in cloth bags until recovery from anaesthesia and released. No mortality or signs of injury were observed following blood meal collection.

We collected opportunistic samples from potential prey and sympatric bat species: one skin sample from a recently dead sea lion *Otaria flavescens* (site L‐10), one pooled sample of human *Homo sapiens* hair from a community shower (site M‐130), two hair samples from cows *Bos taurus* (site M‐134), one hair sample from a great fruit‐eating bat *Artibeus lituratus* (site M‐134) and two hair samples from hairy‐legged vampire bats *Diphylla ecaudata* (M‐134 and AM‐2). Isotopic values for potential prey species or sympatric bats might not be conserved across ecoregions due to isotopic differences in basal resources (Post 2002). However, feeding on livestock can typically be differentiated from feeding on wild prey because most grasses (consumed by livestock) use the C4 pathway (leading to δ^13^C values between −15 and −10‰) (Gannes, Del Rio & Koch 1998) whereas most forest plants use the C3 pathway (leading to δ^13^C between −30 and −25‰). Consequently, the δ^13^C of bat hair can distinguish whether vampire bats fed in the C3 or C4 food webs or both (Voigt & Kelm 2006). Additional prey data could estimate proportional contributions of different prey taxa using isotopic mixing models, but are not necessary to quantify the relative difference in isotopic signatures among individuals within each population and how this varies across populations, which are the focus of this study.

### Stable isotope analysis

Samples for stable isotope analysis were dried at 60 °C for 72 h and cut into small fragments (hair), or ground to a fine powder (skin), following Voigt & Kelm (2006) and Post (2002). For blood meal samples, 3‐mm punches were collected from FTA cards. To differentiate the relative contributions of the blood meal vs. the intrinsic stable isotope values of the FTA card, we analysed blank punches (*N* = 14) from clean areas of FTA cards. δ^13^C signatures were consistent across blank punches (mean = −27·06, SD = 0·13; Fig. S1b), but δ^15^N was highly variable (mean = −0·9, SD = 1·43; Fig. S1a). We therefore excluded δ^15^N blood meal data from further analyses. We used a mixing model to estimate the true δ^13^C value of the stomach contents by calculating the proportional contribution of the blank card to the total isotopic signature (card + stomach contents) and dividing this by the estimated mass of the stomach contents (i.e. [δ^13^C_total_ * Mass_total_/δ^13^C_card_ * Mass_card_]/Mass_blood_) (Phillips & Koch 2002). Isotopic values were expressed in standard δ notation, where δ^13^C or δ^15^N = [(*R*_sample_/*R*_standard_) – 1] × 1000, and *R* is the ratio of ^13^C/^12^C or ^15^N/^14^N). All isotopic analyses used two laboratory standards per 12 samples: bovine (SD = 0·03, 0·00 and mean = 7·49, −21·75, for δ^15^N and δ^13^C, respectively) and spinach (SD = 0·06, 0·08 and mean = −0·5, −27·55, for δ^15^N and δ^13^C, respectively).

### Observed bat bites on livestock and humans

In each site, we recorded the livestock species present and those with evidence of being bitten by bats (Table 1). Prolonged bleeding induced by anticoagulants in vampire bat saliva makes bite wounds easily identified. Bite occurrence was collected qualitatively as presence/absence of bites through discussions with farmers and personal observation. Livestock species presence was based on reports of owners and visual confirmation.

In communities near the two sites in Madre de Dios, we administered standardized questionnaires (*N* = 19) to heads of households to record the incidence of bat bites on humans and the wildlife species present. Questionnaires were conducted in Spanish or Quechua. Following each survey, surveyors assessed the reliability of answers, whether questions were understood, whether the participant was cooperative and the other people present at the interview. Surveys deemed unreliable were excluded.

### Statistical analyses of stable isotope data

We used nested linear models and residual permutation tests to examine differences in isotopic niche positioning among vampire bat colonies following Turner, Collyer & Krabbenhoft (2010). The niche position (i.e. centroid of isotopic space) is considered different if the Euclidean distance between groups is significantly greater than zero (*P‐*value <0·05). The joint distribution of δ^15^N and δ^13^C values (i.e. isotopic niche width) approximates the diversity of feeding strategies among individuals within populations. Isotopic niche width was calculated using the convex hull of δ^13^C and δ^15^N values of individuals in each population and Bayesian ellipse areas (SEA_B_) in the *SIAR* package of r (Jackson *et al*. 2011). The latter approach limits the influence of outliers and enables comparison of populations with unequal sample sizes (Jackson *et al*. 2011). Small values indicate dietary similarity among individuals (i.e. feeding within similar isotopic space) and larger values indicate divergent diets among individuals. We considered non‐overlap in the 95% highest posterior density of estimates as evidence of significant differences among sites (Jackson *et al*. 2011).

To examine the effects age and sex on diet, we applied generalized linear models (GLMs) to the δ^13^C and δ^15^N values of individual vampire bats. Because isotopic baselines could differ among sites, the initial model contained two‐way interactions between site and age and between site and sex and main effects of site, age and sex. For both isotopes, we used a gamma response distribution with a log link, which improved the fit over the Gaussian distribution when comparing the full models by the Akaike Information Criterion (AIC) (δ^13^C: ΔAIC = 41·98, δ^15^N: ΔAIC = 55·79, vs. Gaussian). Model simplification used backwards removal of terms with *P *>* *0·05, selecting for models with the lowest AIC. The statistical significance of differences between pairs of sites in δ^13^C and δ^15^N was tested using the *multcomp* package of R v.3.1.3 (Hothorn, Bretz & Westfall 2008; R Development Core Team 2015).

To evaluate the stability of feeding preferences through time at the colony level, relationships between the range and the average δ^13^C stable isotope values of blood meals (realized prey) and the range and average δ^13^C of hair samples (prey over longer time‐scales) from each colony were tested using Pearson's correlation.

To explore whether provisioning of vampire bats through the introduction of livestock influenced isotopic variability within colonies, we evaluated relationships between the dietary niche width of vampire bat colonies (range of δ^13^C, range of δ^15^N and SEA_B_) and local livestock density using linear regression (α = 0·05). Livestock density estimates included the number of farms with large‐bodied mammalian livestock in the government district of each bat colony (Agricultural Census of Peru, CENAGRO III) and the density of cows, pigs, sheep and goats within a 5, 10 and 20 km radius of each bat colony using modelled livestock density estimates of the Food and Agriculture Organization (FAO, http://www.fao.org/ag/againfo/resources/en/glw/glw_dens.html). Data were processed and assigned to sites using the *raster* package of r (Hijmans & van Etten 2012).

## Results

### The dietary niche of vampire bat colonies across Peru

**Figure 1:**
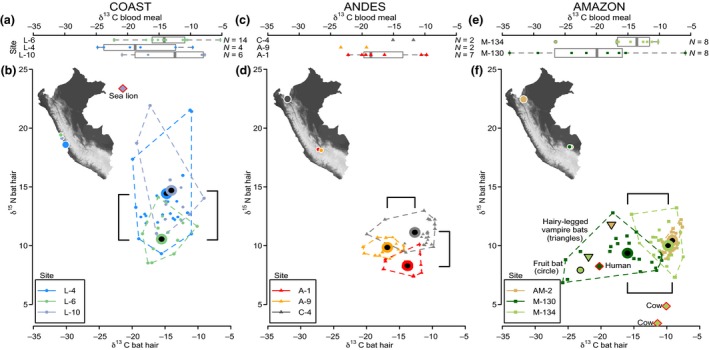
Isotopic variation across sites in potential prey, realized prey and vampire bat hair. Panels a, c and e show boxplots of δ^13^C isotopic values from blood meals collected from vampire bats. Points correspond to each sample analysed, coloured by site; point shapes indicate geographic region (circle = coast, triangle = Andes, square = Amazon). δ^15^N data were excluded because of high background variability in the filter paper used to preserve blood meals. Boxplots are omitted for colonies with <3 blood meals. Panels b, d and f are δ^13^C and δ^15^N data from hair samples from vampire bats; b and f contain data from potential prey (red border) and sympatric non‐*Desmodus rotundus* bats (black border), which serve as reference points for species foraging on forest food resources. Dashed lines are convex hulls of isotopic space. Black points with coloured rings are the centroid isotopic values of each site. Black lines show statistically significant pairwise differences among sites in δ^15^N (vertical bars) and δ^13^C (horizontal bars), analysed independently, using Tukey's honest significant differences (*P *<* *0·001). Statistics for differences between isotopic centroids (i.e. joint distributions of δ^13^C and δ^15^N data) are shown in the main text. Inset maps show sampling locations, with point sizes proportional to the number of hair samples analysed (range = 13–37).

The δ^13^C and δ^15^N of hair samples from *D. rotundus* averaged −13·31 ± 3·74‰ (mean ± standard deviation) and 10·83 ± 2·40‰, respectively; however, pronounced differences existed among regions and among some colonies within regions (Fig. 1b,d,f). Bats from sites located directly on the coastline (L‐4 and L‐10) had significantly higher δ^15^N than the inland site in the same region (L‐6; Fig. 1b), leading to significant differences in the isotopic niche centroids (L‐4 vs. L‐6, *P *=* *0·03; L‐10 vs. L‐6, *P *<* *0·001). Indeed, several individuals in L‐4 and L‐10 showed extreme δ^15^N enrichment – similar to that observed in a hair sample from a sympatric sea lion from L‐10 (Fig. 1b), indicating the presence of bats that routinely feed on animals with high δ^15^N (i.e. predators).

Bats in the Andes had access to different livestock species, but few wild animal prey (Table 1). There, isotopic centroids differed between the three sites (*P *<* *0·002 for all combinations), which arose from differences in both δ^15^N and δ^13^C (Fig. 1d). However, in contrast to the coast and Amazon, bats were more clustered around the centroid isotopic values, indicating greater dietary homogeneity among individuals (Fig. 1d).

In the Amazon, hair samples from bats captured in livestock‐rearing sites (M‐134 and AM‐2) were significantly enriched in δ^13^C relative to bats from M‐130, leading to significantly different centroids of isotopic niche (*P *<* *0·001 vs. M‐130; Fig. 1f). The enriched δ^13^C values of vampire bat hair and blood meals in M‐134 and hair in AM‐2 overlapped with the δ^13^C values of cows in M‐134, suggesting cows as a plausible main food source for these bat populations. Elevated δ^15^N in bats relative to cows is attributed to isotopic enrichment of nitrogen across trophic levels (Post 2002). In contrast, the δ^13^C of vampire bats at M‐130 (where livestock were sparse; Table 1) were depleted to values similar to sympatric bat species that fed in the native forest, suggesting feeding outside of the agricultural food web (Fig. 1f).

### Stable isotope analysis of prey detected in vampire bat blood meals

The range and average δ^13^C stable isotope values of blood meals (realized prey) from colonies were correlated with the range and average δ^13^C of hair samples from bats from the same colony (range bat hair vs. range blood meal: *r *=* *0·61, *P *=* *0·054; average bat hair vs. average blood meal: *r *=* *0·69, *P *=* *0·03). The δ^13^C of bat hair were generally within the range of those observed in the blood meal samples from each respective site, indicating a reasonable characterization of utilized prey resources. For several sites (most notably L‐10 and M‐130), blood meal δ^13^C values were more extreme than those observed in bats, indicating that the species consumed in these blood meals were not the exclusive prey item (Fig. 1a,b,e,f). We expected bimodal distributions differentiating wildlife (C3) from livestock (C4) in blood meals. Instead, prey with intermediate δ^13^C values were also encountered (Fig. 1a,c,e), indicating a wide distribution of isotopic values in the realized prey community on which bats might specialize.

### Variation in the isotopic niche width of vampire bat colonies

**Figure 2:**
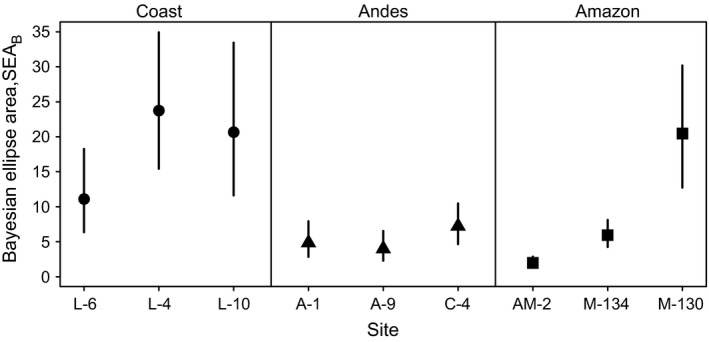
Isotopic niche width measured as the Bayesian ellipse area of δ^13^C and δ^15^N in vampire bat hair samples from nine colonies in Peru. Lines indicate 95% highest posterior densities on the median estimate (black points, with shapes according to region as in Fig. 1). Larger values indicate greater variability in the isotopic values of bats, that is distinct diets among individuals in the same colony.

Different isotopic values within the same population imply consistent differences in feeding among individuals and increase isotopic niche width. We measured the extent of individual heterogeneity in foraging and whether this differed among populations using SEA_B_. Vampire bat colonies in Peru showed over ninefold variation in SEA_B_, with significant variation among sites within the coast and Amazon, but not within the Andes (Fig. 2). The two colonies located directly on the Pacific coastline (L‐10 and L‐4) had considerably larger SEA_B_ than the more inland agricultural colony (L‐6; Fig. 2), which was caused by variability in δ^15^N, that is the trophic positioning of their resource, not δ^13^C (Fig. 1b). In the Amazon, the niche width of M‐130 was 3·4 times greater than that of M‐134 (Fig. 2), caused by a near doubling of the range of δ^13^C values in M‐130 (15·1 vs. 7·8‰, respectively), but similar δ^15^N ranges (5·9 vs. 7·59‰; Fig 1f, Fig. 2), indicating foraging heterogeneity occurred within the same trophic level. The bat colony in the more northern Amazon site (AM‐2) had similar niche width to M‐134, consistent with accessible domestic livestock homogenizing bats’ diets in M‐134 and AM‐2, but not MDD‐130 (Fig. 2).

Generalized linear models revealed effects of site and sex on δ^13^C, whereby males had slightly higher δ^13^C than females (back‐transformed effect size = 1·06 and SE = 1·03, *P *=* *0·04). For δ^15^N, the full model had the lowest AIC which dramatically increased when the site term was removed, whereas removal of other variables and interactions caused less change (ΔAIC from 0·21 to 5·17; Table S1).

### Effects of livestock on bat dietary variability and human depredation

**Figure 3:**
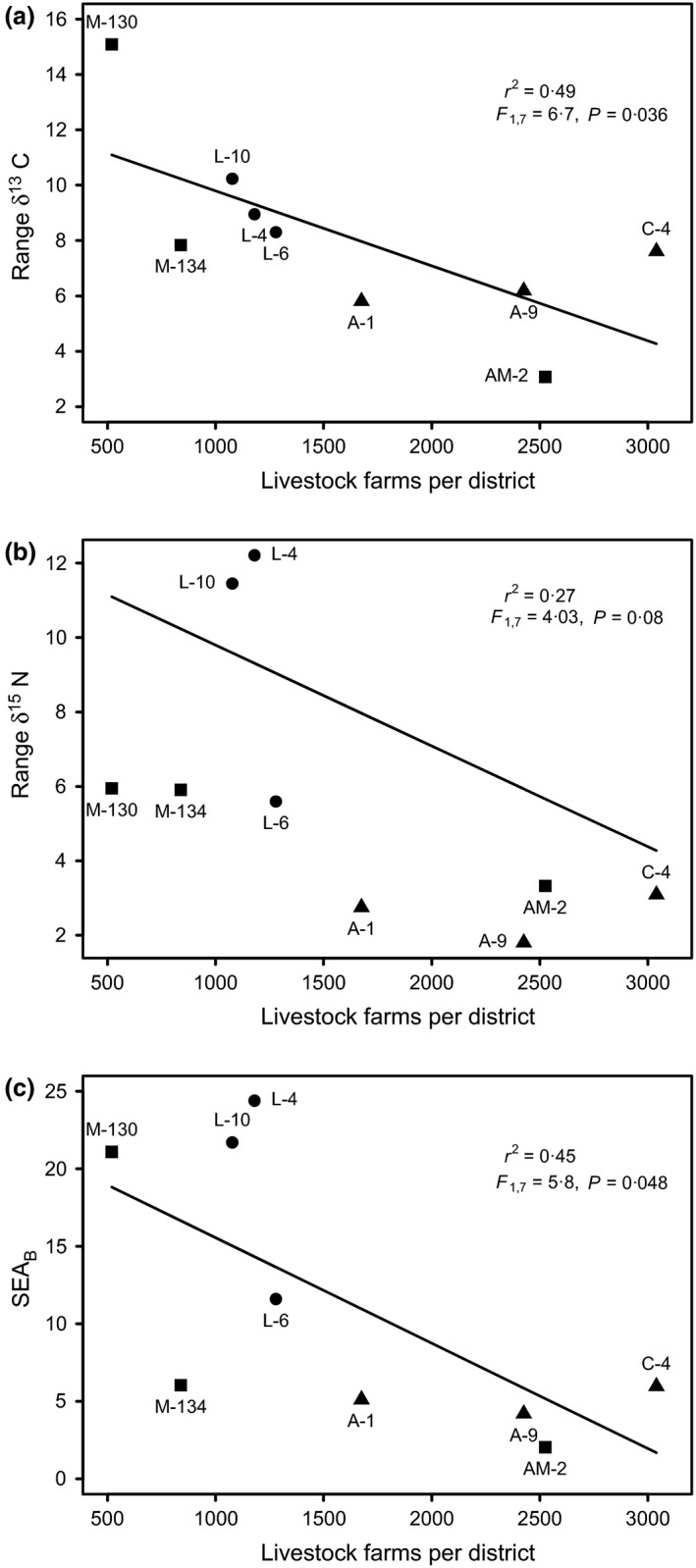
Effects of livestock abundance on isotopic niche width in vampire bats. Measures of dietary diversity included the range of δ^13^C and δ^15^N values in hair samples collected from each colony of vampire bats (a, b) and the Bayesian ellipse area (SEA_B_), estimated from δ^13^C and δ^15^N values in bat hair (c). Points are labelled with site codes; shapes correspond to the geographic region of each colony; see Fig. 1.

The number of farms in the districts (CENAGRO data) where bat colonies were located was negatively correlated with isotopic niche width (Fig. 3a,b,c). Similar relationships occurred with the FAO livestock data, but were only statistically significant after removing an outlier site (AM‐2), where FAO and CENAGRO data conflicted (Fig. S2; SEA_B_ vs. FAO livestock density: *P *=* *0·18, *P *=* *0·06, *P *=* *0·02 for 5, 10 and 20 km radii, respectively). In the Amazon, attacks on humans were not reported in any of *N* = 3 surveys in the livestock‐rearing area in near M‐134, and the local health post (Puesto de Salud, Mazuko) confirmed that bites on humans were extremely rare in the area. In contrast, where livestock were rare in M‐130 (Table 1), vampire bats fed regularly on human beings: nine of fourteen households reported being bitten by bats in the last year with an average of 1·75 bites per household per year (*N* = 21 bites).

## Discussion

The degree to which individuals exhibit specialized diets and how these feeding strategies respond to human activity has drawn attention as an indicator of population‐level responses to environmental change (Bolnick *et al*. 2003; Layman *et al*. 2007b). Previous stable isotope studies have highlighted the tendency for common vampire bats to feed on locally abundant and reliable prey (Voigt & Kelm 2006; Catenazzi & Donnelly 2008; Voigt *et al*. 2008). Yet, the absence of comparable data across colonies with differing prey resources has limited understanding of whether dietary homogeneity arises because vampire bats intrinsically concentrate on local prey that are unanimously favoured or simply feed in habitats with relatively few prey alternatives, limiting opportunities for individual specialization. Thus, the extent to which vampire bats can adapt to new food resources is poorly understood. As vampire bats are the most significant transmitter of rabies in Latin America, this information is critical to anticipate which potential prey species (including humans and livestock) may have high rabies risk in changing environments.

Within single vampire bat colonies, the δ^13^C ranges we observed were among the highest recorded for vertebrates, and the δ^15^N ranges appear unprecedented for single populations of any species, indicating feeding across trophic levels, from primary consumers to predators (Gu, Schelske & Hoyer 1997; Bolnick *et al*. 2003; Araújo *et al*. 2009; Cardona, Aguilar & Pazos 2009). Because of the relatively slow turnover of hair, these differences among individuals can only arise if bats consistently feed on isotopically distinct subsets of the larger prey community. In contrast, inferring the dietary patterns of individuals with similar isotopic values is challenging because feeding on the same prey resource or an ‘averaging out’ of isotopically diverse prey in a generalist diet could theoretically yield the same isotopic value (Peterson & Fry 1987). Our stable isotope analysis of realized vampire bat prey (blood meals) argues against vampire bats as generalist foragers. Specifically, in sites with extreme variation in bat and prey δ^13^C, we also detected prey and bat hair with intermediate δ^13^C (Fig. 1a,c,e). This could enable bats to specialize on prey with intermediate isotopic values in addition to those at the isotopic extremes. Importantly, this information could not be inferred from bat hair alone, and typically requires isotopic data from the potential prey community (Araújo *et al*. 2009), which in our study was unknown, potentially highly diverse and prohibitively challenging to sample directly.

In support of individual heterogeneity in foraging, vampire bats can use memory and/or sensory cues to repeatedly feed on the same individual and form long‐term preferences (Groger & Wiegrebe 2006). Indeed, previous isotopic studies analysing tissues with different isotopic turnover rates (e.g. blood, skin, hair) from the same individuals showed that vampire bat dietary preferences are stable over time (Voigt & Kelm 2006; Voigt *et al*. 2008). The underlying drivers of initial pattern formation in prey selection are less clear. Our GLM analysis found slightly elevated δ^13^C in male bats, but most variation was explained by geographic location, suggesting that simple demographic factors alone are unlikely to drive variation in prey selection. Though food sharing through regurgitation of blood has been documented in vampire bats, it is unlikely to happen often enough to influence stable isotope values (Voigt, Voigt‐Heucke & Schneeberger 2012). Differences among individuals also cannot be explained by prey availability since bats in each site had access to common resources. One possibility is that behavioural factors, such as individual variation in risk aversion (e.g. avoiding open areas where livestock sleep) might contribute to foraging specialization. Though such variation has not been assessed in vampire bats, interindividual variation in boldness was shown in big brown bats *Eptesicus fuscus* (Kilgour & Brigham 2013). Another possibility is that because juvenile vampire bats nurse or forage with their mothers for most of their first year (Greenhall 1988), search images and techniques to feed on specific species may be imprinted. Isotopic studies of mother–pup dyads would be useful to assess maternal components to prey selection.

The accessibility of domestic livestock appeared to reduce the isotopic niche width and therefore the diversity of interspecific contacts of vampire bats (Fig. 3). Within ecoregions, niche width was maximized where both wildlife and livestock were present at low levels, but declined sharply once livestock became abundant. For example, the high niche width of L‐4 and L‐10 can be explained by individuals specializing on both livestock and marine predators (likely sea lions *O. flavescens*), while the absence of the latter in L‐6 truncates niche width. Similarly, niche width was elevated in M‐130 where wildlife, including tapir *Tapirus terrestris*, peccary *Tayassu pecari* and monkeys *Callicebus aureipalatii* and *Saimiri boliviensis*, were accessible, but livestock were rare. Wildlife were also present in M‐134 and AM‐2, but livestock were abundant and highly accessible (<500 m) near bat roosts. Therefore, the fine‐scale accessibility of livestock may be an important determinant of the consequences of livestock on dietary variation among bats. Specifically, we suggest that ecological linkages of vampire bats are maximized under conditions of intermediate disturbance, where livestock are present at low densities in regions with available wildlife prey. Such areas would represent hotspots for interspecific disease transmission at the human–livestock–wildlife interface.

The effects that we observe are unlikely to be driven entirely by variation in isotopic baselines among sites (i.e. same animal, different isotopic value). This is particularly clear in our sites in the Amazon. Sites M‐130 and M‐134 were separated by only 31 km and had similar forest characteristics and elevation, which would be expected to minimize differences in δ^13^C baselines between these sites (McGlynn *et al*. 2009). Yet, bats from these colonies showed essentially no δ^13^C overlap and substantial differences in isotopic niche widths, indicating that differences arise from foraging choices not variation in baseline isotopic levels of the same prey species (Table 2; Figs 1 and 2). Similar niche differentiation over small spatial scales was observed in lemur communities in Madagascar (Dammhahn & Kappeler 2014). Both in the Amazon and on the coast, finding vampire bats with isotopic values similar to other wildlife that do not exploit anthropogenic food resources (fruit bats, sea lions, etc.) further supports bats switching between food webs when livestock are rare.

**Table 2:** Uncorrected measures of isotopic niche width within vampire bat colonies. Standard ellipse area (SEA) was estimated using the *standard.ellipse* function in the *SIAR* package of r. Sample sizes are provided in Table 1

Colony	δ^13^C range	δ^15^N range	SEA	Convex hull area
A‐1	5·81	2·75	4·72	10·12
A‐9	6·18	1·80	3·62	7·81
AM‐2	3·07	3·32	1·71	4·87
C‐4	10·35	3·55	7·32	24·23
L‐10	10·24	11·45	22·79	56·71
L‐4	8·95	12·21	25·16	80·80
L‐6	8·30	5·59	11·92	26·34
M‐130	15·09	5·95	21·47	51·66
M‐134	7·84	5·91	6·01	28·71

The shift to a livestock‐based diet poses interesting questions for vampire bat health and behaviour. Blood from domestic species might affect vampire bats directly through differences in nutritional quality, through exposure to new pathogens and antibiotics, or indirectly through altering gut microbial communities, which are increasingly appreciated to influence a wide variety of processes (Ezenwa *et al*. 2012). Foraging strategies in vampire bats also have direct implications for rabies transmission. The feeding patterns we describe raise a strong possibility for transmission from bats to wildlife in the Amazon and along the Pacific coast, which could establish new rabies reservoirs or create intermediate hosts for human infections. Such transmission events have caused prolonged epizootics in wildlife and are recognized as a public health and veterinary threat throughout the Americas (Kuzmin *et al*. 2012).

Human rabies risk is speculated to increase following land‐use change because extirpation of wildlife by hunting or deforestation could increase feeding on humans (Schneider *et al*. 2009; Stoner‐Duncan, Streicker & Tedeschi 2014). In support of this hypothesis, bats from colony M‐130 in the Amazon fed on both wildlife (as suggested by our stable isotope analysis) and humans (as indicated by questionnaires), indicating the possibility for dietary switching. Genetic analyses of the species origins of blood meals could assess whether humans and bats compete for the same wildlife prey, such that hunting increases rabies risk by depleting preferred vampire bat prey. Finally, our data are consistent with the hypothesized zooprophylactic effect of livestock in reducing depredation on human beings (Gilbert *et al*. 2012), as bites were common in Amazonian communities without livestock, but absent in communities that had cattle.

In summary, we show that the diversity of foraging strategies adopted by individual vampire bats is context‐dependent and likely influenced by both the inherent diversity of sympatric prey and anthropogenic factors that alter prey availability. Improved knowledge of the role of individuals for population, community and ecosystem dynamics is critical to inform strategies to manage animal conservation but also public health priorities arising from wildlife pathogens, as is the case in our study region. Our findings provide a novel perspective on this topic, demonstrating that remarkable variability in dietary specialization can transform to dietary homogenization in rapidly changing ecological communities with consequences for altered interspecific contact rates and disease transmission.

## Ethics statement

The University of Georgia's Institutional Animal Care and Use Committee approved protocols for the capture and handling of bats (AUP # A2009‐10003‐0), and collection and exportation permits were granted from the Peruvian authorities (RD‐222‐2009‐AG‐DGFFS‐DGEFFS; RD‐273‐2012‐AG‐DGFFS‐DGEFFS; 003851‐AG‐DGFFS). The Human Subjects Office of the Institutional Review Board of the University of Georgia approved questionnaire procedures (Project # 2009‐10832‐1).

## Data accessibility

Stable isotope data, sample locations and survey data are uploaded as online supporting information (Appendices S1 and S2).

## Supporting information


**Fig. S1.** Comparison of uncorrected δ^13^C and δ^15^N values in blank punches from FTA cards versus cards plus samples of stomach contents.

**Fig. S2.** Comparison of FAO data calculated at 3 radius distances and Peruvian census (CENAGRO) data on livestock densities.

**Table S1**. Summary of generalized linear models of ecological correlates of stable isotope values.

**Appendix S1.** Stable isotope values for bat hair samples collected in the study (comma‐separated text file).

**Appendix S2.** Stable isotope values for blood meal samples collected from engorged bats (comma‐separated text file)
