# Supplementary material for: Foraging choices of vampire bats in diverse landscapes: potential implications for land‐use change and disease transmission
Source: J Appl Ecol. 2016 May 26;53(4):1280–8. doi: 10.1111/1365-2664.12690 (PMC4950014; doi:10.1111/1365-2664.12690)

Figure S1. Comparison of uncorrected $\delta^{13}C$ and $\delta^{15}N$ values in blank punches from FTA cards versus cards plus samples of stomach contents.

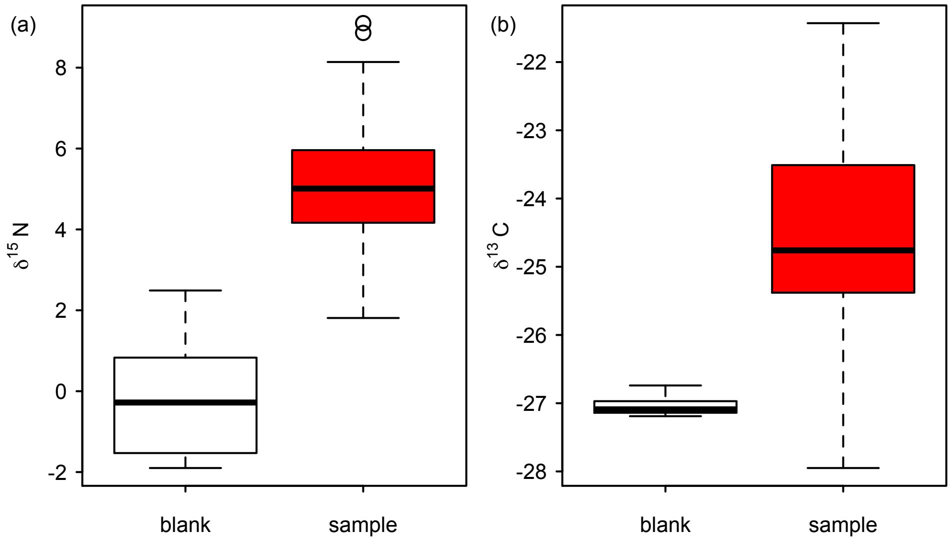

Supplement: Supplementary file 1 — Fig. S1. Comparison of uncorrected δ13C and δ15N values in blank punches from FTA cards versus cards plus samples of stomach contents. [file JPE-53-1280-s001.pdf]
