# Supplementary material for: Foraging choices of vampire bats in diverse landscapes: potential implications for land‐use change and disease transmission
Source: J Appl Ecol. 2016 May 26;53(4):1280–8. doi: 10.1111/1365-2664.12690 (PMC4950014; doi:10.1111/1365-2664.12690)

Figure S2. Comparison of FAO data calculated at 3 radius distances and CENAGRO data on livestock densities. Colors correspond to those indicated in Figure 1 of the main text. Note the density of livestock at AM-2 as consistently underestimated by FAO data across all panels.

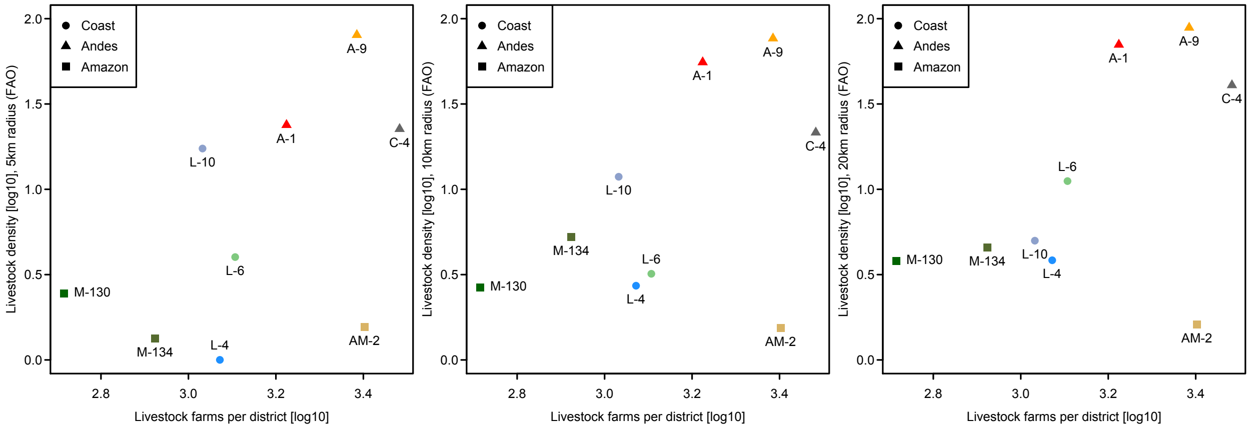

Supplement: Supplementary file 2 — Fig. S2. Comparison of FAO data calculated at 3 radius distances and Peruvian census (CENAGRO) data on livestock densities. [file JPE-53-1280-s002.pdf]
