# Supplementary material for: Foraging choices of vampire bats in diverse landscapes: potential implications for land‐use change and disease transmission
Source: J Appl Ecol. 2016 May 26;53(4):1280–8. doi: 10.1111/1365-2664.12690 (PMC4950014; doi:10.1111/1365-2664.12690)
Supplement: Supplementary file 3 — Table S1. Summary of generalized linear models of ecological correlates of stable isotope values. [file JPE-53-1280-s003.docx]

| **δ^13^C** | **Model** | **d.f.** | **∆AIC** |
| --- | --- | --- | --- |
|  | site+sex | 11 | 0 |
|  | site | 10 | 1.8116 |
|  | site+sex+age | 12 | 2.0739 |
|  | site+site*sex+age | 20 | 11.1409 |
|  | site+site*sex+site*age | 24 | 15.8564 |
|  | age+sex | 4 | 146.2731 |
| **δ^15^N** | site+site*sex+site*age | 24 | 0 |
|  | site+site*sex+age | 20 | 0.2073 |
|  | site | 10 | 2.9127 |
|  | site+sex | 11 | 4.5151 |
|  | site+sex+age | 12 | 5.1661 |
|  | age+sex | 4 | 133.7937 |

**Table S1.** Summary of generalized linear models tested to explain variation in δ^13^C and δ^15^N.
